# Development and Application of Nanoparticle-Nanopolymer Composite Spheres for the Study of Environmental Processes

**DOI:** 10.3389/ftox.2021.752296

**Published:** 2021-12-13

**Authors:** Robert J. Rauschendorfer, Kyle M. Whitham, Star Summer, Samantha A. Patrick, Aliandra E. Pierce, Haley Sefi-Cyr, Soheyl Tadjiki, Michael D. Kraft, Steven R. Emory, David A. Rider, Manuel D. Montaño

**Affiliations:** ^1^ Department of Environmental Sciences, Western Washington University, Bellingham, WA, United States; ^2^ Department of Chemistry, Western Washington University, Bellingham, WA, United States; ^3^ Postnova Analytics Inc., Salt Lake City, UT, United States; ^4^ Scientific Technical Services, Western Washington University, Bellingham, WA, United States; ^5^ Department of Engineering and Design, Western Washington University, Bellingham, WA, United States

**Keywords:** microplastics, single particle ICP-MS, core-shell, estuarine sediment, tracers

## Abstract

Plastics have long been an environmental contaminant of concern as both large-scale plastic debris and as micro- and nano-plastics with demonstrated wide-scale ubiquity. Research in the past decade has focused on the potential toxicological risks posed by microplastics, as well as their unique fate and transport brought on by their colloidal nature. These efforts have been slowed by the lack of analytical techniques with sufficient sensitivity and selectivity to adequately detect and characterize these contaminants in environmental and biological matrices. To improve analytical analyses, microplastic tracers are developed with recognizable isotopic, metallic, or fluorescent signatures capable of being identified amidst a complex background. Here we describe the synthesis, characterization, and application of a novel synthetic copolymer nanoplastic based on polystyrene (PS) and poly(2-vinylpyridine) (P2VP) intercalated with gold, platinum or palladium nanoparticles that can be capped with different polymeric shells meant to mimic the intended microplastic. In this work, particles with PS and polymethylmethacrylate (PMMA) shells are used to examine the behavior of microplastic particles in estuarine sediment and coastal waters. The micro- and nanoplastic tracers, with sizes between 300 and 500 nm in diameter, were characterized using multiple physical, chemical, and colloidal analysis techniques. The metallic signatures of the tracers allow for quantification by both bulk and single-particle inductively-coupled plasma mass spectrometry (ICP-MS and spICP-MS, respectively). As a demonstration of environmental applicability, the tracers were equilibrated with sediment collected from Bellingham Bay, WA, United States to determine the degree to which microplastics bind and sink in an estuary based of grain size and organic carbon parameters. In these experiments, between 80 and 95% of particles were found to associate with the sediment, demonstrative of estuaries being a major anticipated sink for these contaminants. These materials show considerable promise in their versatility, potential for multiplexing, and utility in studying micro- and nano-plastic transport in real-world environments.

## Introduction

Plastic pollution is a pervasive ecological concern brought on by the ubiquity of plastics and their recalcitrance to environmental degradation (Claessens et al., 2011; Law and Thompson, 2014; Rochman, 2018). In recent years microplastics (MPs), defined as plastic material <5 mm, have been identified as a contaminant of concern capable of transport and deposition in wide array of environments from urban rivers (McCormick et al., 2014; McCormick et al., 2016) to Arctic coasts (Anderson et al., 2016) and remote mountain catchments (Allen et al., 2019). MP entry into the environment can occur through a number of intentional and unintentional release pathways contingent on the product life cycle (Steensgaard et al., 2017). Polyester fibers commonly found in textiles typically concentrate in the biosolids of wastewater treatment plants (Hernandez et al., 2017), while polystyrene plastic debris often deposit and collect in urban streams and landfills (Davis and Murphy, 2015; Lu et al., 2016). Moreover, the toxicity of these materials is not well understood, and a particular concern is their capacity to adsorb and transport hydrophobic persistent organic pollutants (POPs). Understanding the role MPs may play as vectors for POPs and hydrophobic organic contaminants (HOCs) is a particular challenging prospect as it requires an understanding the relative rates of partitioning between the polymer, the organic contaminant, and the biotic ligand (i.e. tissue). It should be noted, as these studies continue to be refined, evidence increasingly suggests that MP vector transport might not be a significant contributor to their risk (Koelmans et al., 2016; Koelmans et al., 2021).

Understanding MP fate, transport, and toxicity requires analytical methods and instrumentation with the requisite sensitivity and selectivity to parse out MP signatures within complex biological and environmental matrices. Many established methods rely on separation (e.g., filtration, sieving, sedimentation) followed by physical identification via light microscopy, which has inherently limited size resolution (Brander et al., 2020). More sophisticated methods may utilize spectroscopic techniques like Fourier transform infrared (FTIR) spectroscopy or Raman microscopy that permit chemical specific identification based on the molecular structure of the polymeric monomers (Song et al., 2015; Shim et al., 2017). However, these techniques are similarly limited by their size resolution, only capable of identifying MPs down to the 1 μm length scale. Mass spectrometry, particularly pyrolysis gas chromatography mass spectrometry, has been gaining traction as an appropriate technique for MP analysis, but excludes size and number information pertinent for assessing environmental behavior (Fries et al., 2013; Mitrano et al., 2021). With the increasing recognition of nanoplastic (1–100 nm length scale) concentrations in the environment, it is evident that new analytical methods are needed to assess MP behavior with the requisite sensitivity in complex systems.

One means of studying MP fate and behavior in environmental systems is through the development of MP tracers; materials that behave like MPs but contain identifiable elemental or fluorescent components not commonly found in environmental matrices. The use of fluorescent markers in particles has seen widespread use, particularly in assessing MP biological uptake and consumption (Karakolis et al., 2018; Johnson et al., 2021) and transport in aquatic systems (Cook et al., 2020). However, there are concerns regarding the leachability of these additives, as well as their photolability in natural environmental systems (Luo et al., 2019). Other potentially more stable methods are to use labeled carbon-13 MP or radiolabeled carbon-14 materials, but these may require high cost or specialized equipment for detection (Zumstein et al., 2018; Sander, 2019; Sander et al., 2019). Still another approach is to include metallic signatures and nanoparticles within the polymer matrix, that can then be detected using techniques such as ICP-MS (Mitrano et al., 2019). This approach has allowed MP behavior to be studied in complex matrices ranging from the livers of freshwater mussels (Facchetti et al., 2020) to the complex mixture of wastewater sludge (Keller et al., 2019; Schmiedgruber et al., 2019). These studies have shown great promise in examining the behavior of MPs and providing information on the fate, behavior, and transport of MPs in environmental release scenarios.

In this study, we build upon this work to develop a core-shell polymer platform the allows for the development of multiple potential MP tracers that can be characterized and quantified by single particle ICP-MS (spICP-MS) (Montano et al., 2016). Single particle-based methods have recently been applied in monitoring polymeric degradation (Barber et al., 2020) and also in tracking carboxylated microspheres (Jiménez-Lamana et al., 2020), but has yet to be applied in an environmental context for MP analysis. The core polymeric material is a polystyrene-poly(2-vinylpyridine) co-polymer (PS-co-P2VP) which had been previously shown to be a suitable platform for the attachment and growth of metallic nanoparticles (Curtis et al., 2018). These nanoscale polymer core particles were then overcoated with additional polymeric material (polystyrene or polymethylmethacrylate) to ensure that their behavior and interactions with environmental substrates mimicked that of ‘pristine’ MP particles. These nanoparticle-polymer tracers (NP-Tracers) can then be used to study the behavior of MPs in environmental systems while being quantified and characterized by their metallic signatures. As a proof-of-concept, the aggregation and settling behavior of these NP-Tracers in estuarine sediment collected from Bellingham Bay, Washington, United States. The application of these particles to real-world environmental matrices affords the ability to better understand MP and nanoplastic behavior, potentially leading to a better understanding of their ultimate environmental behavior and may be used to better regulate MP materials going forward.

## Methods and Materials

### Nanoparticle-Polymer Tracer (NP-Tracer) Preparation

The core material synthesis (PS-co-P2VP) has been previously described elsewhere (Curtis et al., 2018). Briefly, an emulsion polymerization approach was used, whereby a poly(ethylene glycol) methacrylate (PEGMA) stabilizer, surfactant (Aliquat 336), and cross-linker (divinylbenzene) were held constant and in proportion to the total molar amount of the remaining monomers, styrene and 2VP. The divinylbenzene is a key component, acting as a cross linker binding the polymer core together and allows the particle to swell in size and return to its initial size when exposed to low pH and neutral pH conditions, respectively. Prior to gold ion loading (with 24 mM KAuCl_4_ in aqueous HCl at pH = 2.5), polymer cores are shelled with the polymer of interest (herein MMA or PS) via a seeded emulsion polymerization approach (Li et al., 2019). In brief, three solutions were preparedand combined according to the following steps. The first solution consisted of PS-co-P2VP polymer cores (5.0 ml; 8.5 wt% polymer cores suspended in water) and 0.01 g of Aliquot 336. The second solution contained ammonium persulfate (0.03077 g) and 4.0 ml of ultrapure water (Barnstead Nanopure Purifier; resistivity of 18 MΩ cm). The third solution contained 11 ml of ultrapure water, shell monomer (MMA or S; 1.0 ml), and 0.01 g of Aliquot 336. A polymerization reaction flask was loaded with the first solution and immersed into a heated oil bath set to 80°C. One third of the second solution was added to the reaction flask, while the remaining two thirds of the solution were loaded into a syringe and syringe pump. The syringe pump delivered the remaining contents dropwise into the reaction flask over a 3.5 h period. Simultaneously, the third solution was loaded into a separate syringe and syringe pump that was programmed to deliver its contents to the reaction flask over the same period of time. After complete delivery of both solutions, the shell polymerization reaction solution was allowed to stir for an additional 30 min followed by an ice bath quench. When Pd or Pt NP-Tracers are desired instead, the above 24 mM KAuCl_4_/HCl solution is replaced with 23 mM KPdCl_6_/HCl and 20 mM KPtCl_6_/HCl equivalents for ion loading.

**FIGURE 1:**
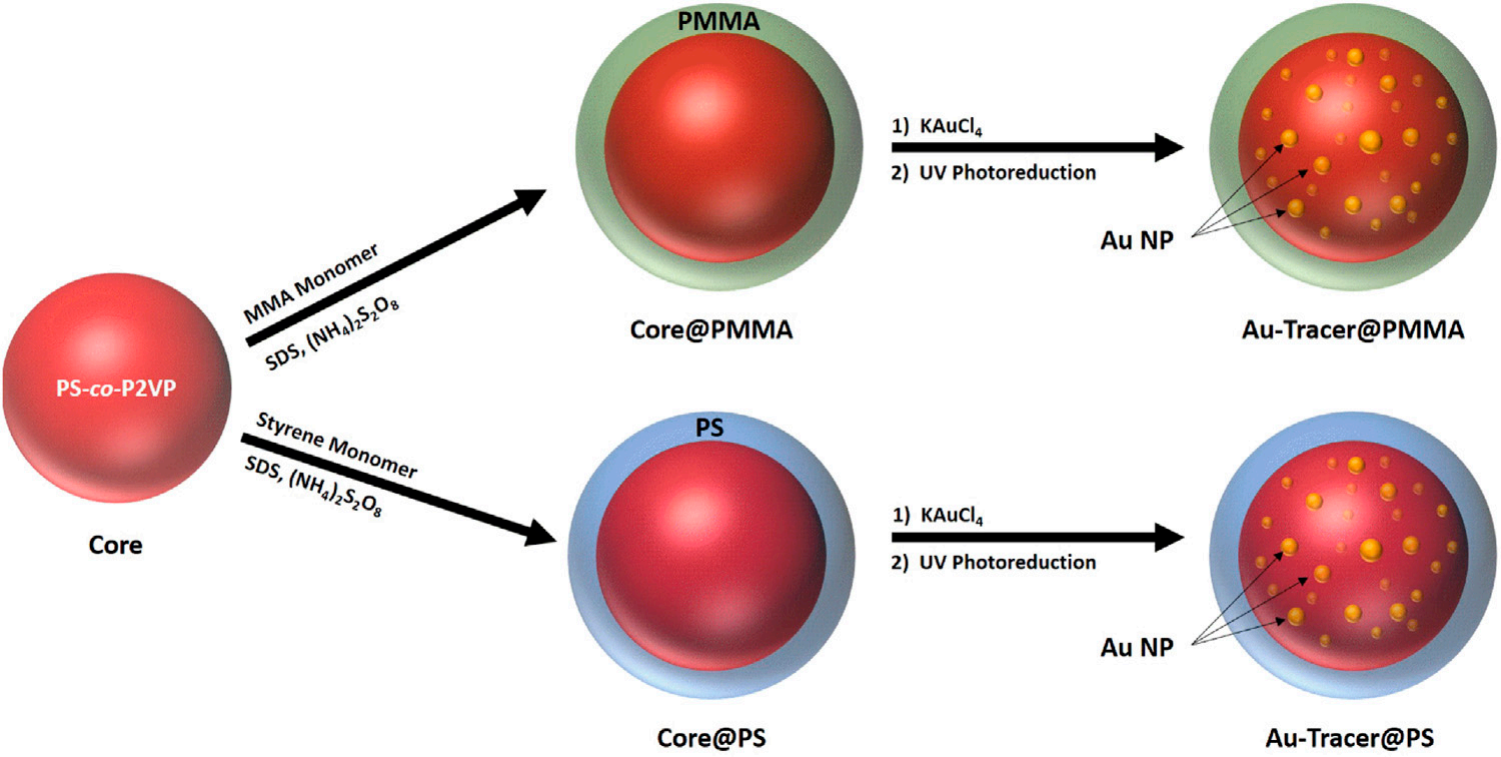
Synthesis scheme for Au-Tracer@PMMA and Au-Tracer@PS particles.

Shelled polymer cores (core@shell) were purified by dialysis (4-5 sequential 4 h ultrapure water dialysis steps with MWCO of 12 ,000–14 ,000 membrane). The polymerized 2VP units in the polymer core allow for the electrostatic stabilization of the gold chloride ions that are loaded into the core and anchors the ions electrostatically during Ultraviolent (UV)-triggered photoreduction with in a UV chamber. The solution then underwent a stirred photoreduction reaction for 18 h using a Rayonet model RPR-600 UV photochemical reactor equipped with eight light sources, each with a 253.7 nm maximum emission and a power of 8 W. The resulting Au NP-Tracers were purified by three sequential 8 h ultrapure water dialysis steps (MWCO of 12,000–14,000 kDa membrane). Photoreduction resulted in solid gold particles that grow inside the core of the nanoplastic, ensuring that the gold does not leach out into the solution. A representation of the synthetic process is shown in Figure 1.

### NP-Tracer Physical Characterization

The NP-Tracers were characterized using multiple nanoanalysis techniques to ensure that particles were of a consistent size distribution, composition, and had physical and chemical characteristics that mirrored that of their pristine counterparts. The size of the NP-Tracers was characterized using dynamic light scattering (DLS, Delsa Nano HC, Beckman Coulter), tapping-mode Atomic Force microscopy (AFM, Bioscope Catalyst, Bruker), and high-angle annular dark-field (HAADF) imaging using Deben scanning transmission electron microscope with a 30 keV beam(STEM; JSM-7200F field emission microscope, JEOL). The STEM and AFM analysis were used to determine the extent of nanoparticle incorporation in the core of the tracer material. SEM-EDX was also performed to examine the incorporation of Au into the tracer particles.

The hydrodynamic diameter of the Au-Tracer@PS particles were also analyzed by asymmetric flow field flow fractionation (AF4, AF 2000, Postnova Analytics) coupled to UV-vis and the density of the particles measured using sedimentation flow field fractionation (Sed-FFF, CF 2000, Postnova Analytics) with the operating conditions presented in Supplementary Tables S1–S3. These measurements were performed in comparison to a 510 nm polystyrene latex standard (Thermo Fisher Scientific) and a 499 nm PMMA standard (microParticles GmbH).

Zeta potential measurements were performed for a select number of aquatic conditions for the Au-Tracer@PS particles. These measurements were performed using a Malvern Zetasizer (Malvern Panalytical).

### NP-Tracer Chemical Characterization

Due to the complex nature of the core@shell NP-Tracer platform, the tracer particles were broadly characterized by Raman, Fourier-transform infrared spectroscopy (FTIR), and thermogravimetric analysis (TGA) at each step of the synthesis process. A key need was ensuring that the exterior of the NP-Tracer had been adequately over-coated with the intended polymer, therefore samples of the NP-Tracers before and after ‘shelling’ (the addition of the external polymer label) were analyzed by FTIR in reflectance mode (Nicolet iS10 FT-IR spectrometer), TGA (TA Instruments Q500).

For Raman imaging and analysis, Core, Core@PS, and Core@PMMA samples were prepared by pipetting 5 μL of the respective material on a dry glass cover slip (No. 1, 25 mm^2^, Corning Glass). The sample was allowed to dry for 2 h before analysis. Raman spectra were acquired with a Renishaw inVia Qontor Raman microscope using parameters shown in Supplementary Table S4. Polystyrene standards for spectroscopic analysis were prepared with 10 μm Polystyrene beads (Polysciences, 99%) and PMMA standards prepared with 3–5 mm PMMA pellets (Nanoshel, 99.9%).

Lastly, it was important to test the ‘ruggedness’ of the tracers and evaluate the potential for the Au NPs to leach from the Au-Tracer platform. In order to address this, Au-tracer@PS particles were spiked into the three different aquatic media to be tested: Ultrapure water, EPA Moderately Hard water, and 30 g L^−1^ Instant Ocean Saltwater. These samples were allowed to sit for 48 h and then filtered through a 0.22 μm mixed cellulose ester syringe filter and acidified with a 1:4 mixture of HNO_3_/HCl solution for 24 h prior to analysis by ICP-MS. This was additionally performed with a freshly prepared stock of Au-Tracer@PS particles for comparison to evaluate any potential for Au NP leaching (Supplementary Figure S1).

### Single Particle ICP-MS

A key characterization and quantification technique used in this study is single particle ICP-MS (spICP-MS) which enables the counting and sizing of engineered nanomaterials (Montano et al., 2016). The inclusion of gold NPs in the NP-Tracers allows the detection of the nanoplastic material by proxy of the gold NP signal events. As each NP-Tracer enters the plasma of the ICP-MS, a plume of gold ions is created and detected as a single event. These events represent the number of NP-Tracers in the system being analyzed.

For this study, spICP-MS was carried out using a quadrupole ICP-MS (Agilent 7500ce), with a MicroMist glass nebulizer and a Quartz Scott-type spray chamber with typical instrument operating conditions described in the Supplementary Table S5. For spICP-MS applications, instrument detection operated at dwell times of 10 ms. Transport efficiency was measured using the mass-based method (Pace et al., 2011) with a nominal 60 nm gold nanoparticle used for the size-based standard (Nanocomposix). All dissolved standards were prepared in 2% trace-metal grade nitric or 2% trace-metal grade hydrochloric acid (Fisher Scientific) with ultrapure water (18.2 MΩ) used for any subsequent dilutions (EMD Millipore).

The most important parameter measured by spICP-MS in this study was particle number concentration. These values were calculated from spICP-MS measurements using the following equation:

$$\frac{Particles}{mL}=\frac{\left(\#\;of\;events\right)}{\eta_{neb}}*\frac{DF}{Q_{flow}}$$ (1)

Where the ratio of the number of events are the number individual particle signals measured by spICP-MS (i.e., the intensity spikes above the background in Figure 4A) and the transport efficiency (η_neb_, i.e., nebulization efficiency) provides the number of particles in the volume of sample analyzed. The transport efficiency multiplied by the dilution factor (DF) and the sample flow rate (Q_flow_) provides a particle number concentration in particles mL^−1^ (Pace et al., 2011).

### Estuarine Sampling Site Collection, Characterization and Analysis

Estuaries are an ideal site for the study of microplastic behavior. The relatively abrupt change in aquatic chemistry, going from freshwater to saltwater, has significant implications for the transport and behavior of colloidal systems (i.e., microplastics, nanoparticles). As such, it is likely that coastal areas and estuaries will serve as sinks and accumulation areas for any MP material transported via freshwater streams (Auta et al., 2017; Bessa et al., 2018). The degree to which MPs will settle and accumulate in these areas will depend on hydrodynamic forces in the bay, the aquatic chemistry of the estuary, and the composition of the sediment.

Sediment was collected from Bellingham Bay, WA, United States to be used in sediment equilibration experiments with the NP-Tracers. Northern Bellingham Bay is an estuary formed from the Nooksack river outlet meets the marine water from the Salish Sea. Sediment samples were collected at low tide August 2020. Seven sites were selected that encompassed an area of 2.45 km^2^ and top 10 cm of sediment was collected in triplicate using a sediment core sampler, a stainless-steel trowel, and a plastic container at each site (Map of sampling sites shown in Supplementary Figure S2).

Prior to equilibration experiments, the sediment grain size, metal content, and carbon content were quantified for each site (Supplementary Tables S6, S7). For the sediment grain size, each sample was placed in an oven at 60°C for a minimum of 24 h and the mass of the dried sediment was obtained. The sediment was passed through a stainless-steel sieve with mesh sizes of 2000, 500, 250, 125, and 63 µm. A top and bottom cover were placed on the sieve stack and the entire apparatus was manually shaken for a summed total of 15 min. The mass of each fraction was obtained, and the grain size percentage was calculated from the total mass (Supplementary Figure S3 and Supplementary Table S7).

Sediment metal concentrations were determined after drying and digestions EPA Method 3050B (Martin et al., 1994). Trace metals were analyzed via ICP-MS (Agilent 7500ce) following EPA Method 200.8 (USEPA, 1994b) and the major metals were analyzed by FAA (Varian Spectra AA 220Z) following EPA Method 7000B. For trace metal analysis, a calibration standard (ICP-MS-6020-CAL-R-1, AccuStandard) containing Al, V, Mn, Co., Ni, Co., Ni, Cu, Zn, and Cd was used to create and external calibration curve via serial dilution in 2% trace metal grade nitric acid.

To ensure that the gold NPs associated with the PS tracers (Au-Tracer@PS) would be a suitable metallic fingerprint, the tracer gold concentration of the sediment was determined through acid digestion followed by ICP-MS detection. Sediment digestion used 5 ml aqua regia solution (4:1 concentrated hydrochloric acid and concentrated nitric acid) added to 1 g of dried sediment. The mixture was refluxed for 1 hour, left to cool overnight, washed and filtered before being brought to a 50 ml volume using ultrapure water. The samples were analyzed using the ICP-MS calibrated using a dissolved gold standard (SPEX Certiprep).

Carbon speciation of the sediment samples followed a chemo-thermal oxidation (CTO) method that differentiated inorganic, labile, and black carbon sources. The method performed is an adaptation of the following methods (Gustafsson et al., 2001; Elmquist et al., 2004; Caria et al., 2011). The sediment fractions were analyzed using an elemental analyzer (CE Elantech EA 1112) that determined the percent carbon associated with each sample (Supplementary Table S6).

### Environmental Application Experiments

In order to demonstrate the potential environmental application of the NP-Tracers, two different experiments were developed to assess their aggregation and settling behavior. In the first, two different types of aquatic media were prepared: an EPA mod hard water to represent a freshwater (Weber, 1991) and a 30 g L^−1^ saltwater (Instant Ocean) media (Schierz et al., 2012).

To investigate the behavior of the NP-Tracers in an estuarine environment, a sediment slurry was created from sediment collected at each site. These slurries were then spiked with a predetermined amount of NP-Tracers for a final concentration of 1.5 × 10^8^ particles mL^−1^. The slurry was then allowed to equilibrate via a table shaker over the course of 48 h, after which samples were allowed to settle for 40 min, before sampling from the top 2 cm of the vial for spICP-MS analysis, representing an ‘unbound’ fraction of NP-Tracers. These experiments were repeated for each sediment site in both EPA moderately hard water and 30 g L^−1^ saltwater generated from dissolved Instant Ocean in ultrapure water.

### Statistical Analysis and Graphing

All statistical analysis and graphing were performed using OriginPro 2020b version.

## Results

### Physical Characterization of NP-Tracer Particles

The cores, shelled cores (core@shell), and tracer particles were characterized by a number of different sizing techniques. Measured sizes are reported in Table 1 (DLS for Au-Tracer@PMMA in Supplementary Figure S4). Particle diameters measured by SEM and AFM are reported as a mean and standard deviation of 25 recorded particle sizes. DLS measured diameters are given as the average and width of the distribution for the measured particles. The stock particle number concentration was determined via spICP-MS according to methodology set out by Pace et al. (2011). Briefly, the measured number of events are divided by the transport efficiency and the flow rate to determine the number of particles in a given volume. This determination was made at three different dilution factors, which were all in agreement (Supplementary Figure S5).

**TABLE 1:** Measured sizes of core, shelled, and tracer materials used in this study. Stock particle number concentrations were determined using single particle ICP-MS. Data represents the average and standard deviation, excepting DLS where the error represents the width of the size distribution.

Sample	SEM size (nm)	DLS size (nm)	AFM size (nm)	Particle no. conc. (Particle mL^−1^)
Core	325 ± 11	367 ± 28	269 ± 15	^a^
Core@PS	457 ± 13	441 ± 47	442 ± 51	^a^
Core@PMMA	364 ± 13	404 ± 97	349 ± 26	^a^
Au-Tracer@PS	477 ± 25	446 ± 195	480 ± 26	2.65(±0.28) × 10^11^
Au-Tracer@PMMA	389 ± 22	494 ± 71	390 ± 55	5.31(±0.20) × 10^11^

a Samples not measured as there is not detectable metal loading for spICP-MS.

**FIGURE 2:**
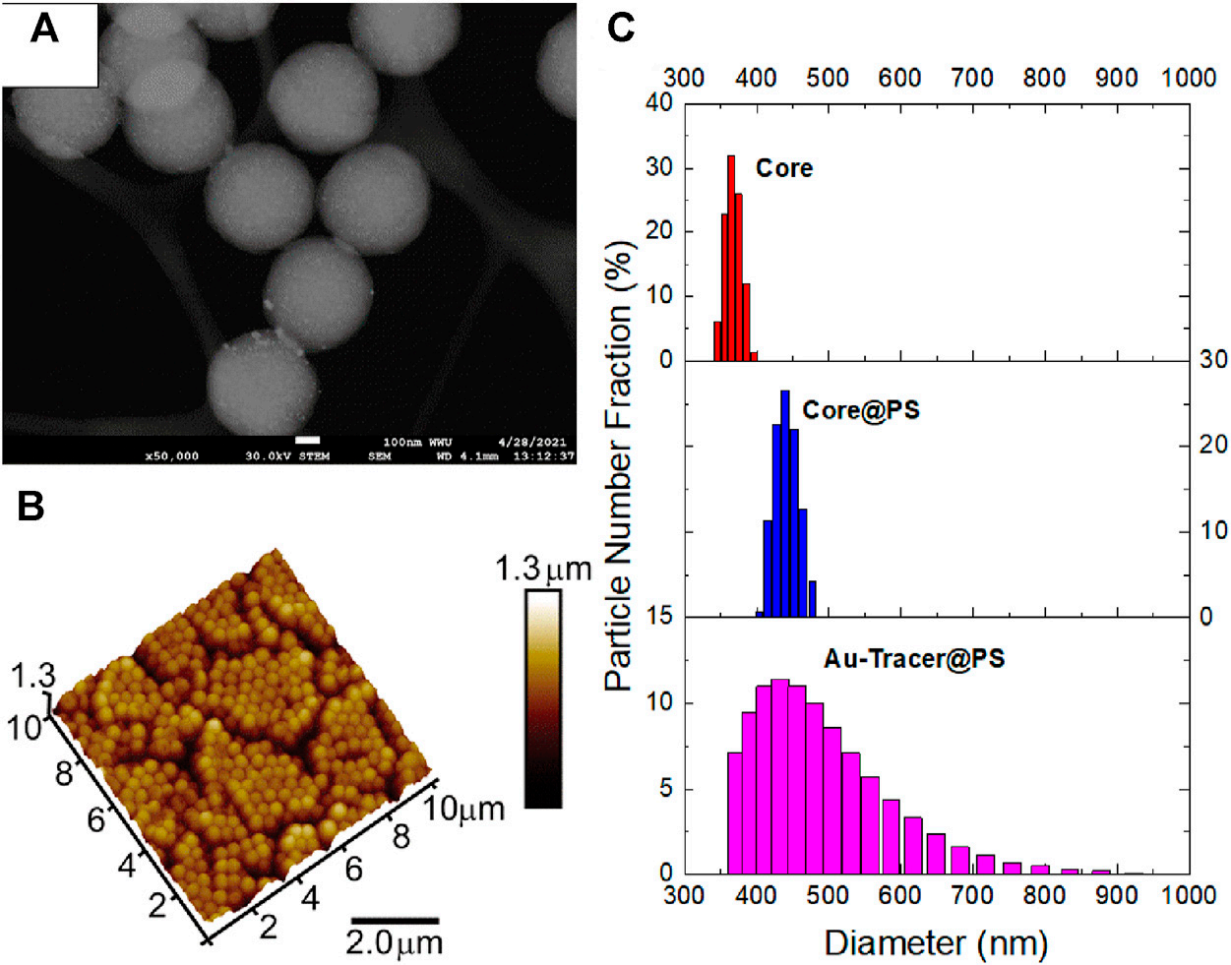
Physical characterization of Au-Tracer@PS particles. **(A)** SEM images of Au-Tracer@PS particles. **(B)** Tapping-MAFM image of Au-Tracer@PS particles. **(C)** DLS data detailing the core size **(top)**, core@PS size **(middle)**, and Au-Tracer@PS size **(bottom)**.

Examples of particle sizing are shown in Figure 2. Figure 2A is an SEM image showing an Au-Tracer@PS. Figure 2B is an AFM analysis of Au-Tracer@PS material. Figure 2C are the size distributions as determined by DLS for the core, shelled, and tracer material. Additional SEM-EDX mapping images were taken of the Au-Tracer@PS material to further demonstrate the inclusion of gold nanoparticles inside the polymer matrix (Supplementary Figure S6). The size of the material Au-Tracer@PS material was also determined by AF4-UV-vis in comparison to two polystyrene standards (Supplementary Figure S6). The particle density was determined by sedimentation-FFF, which separates particles according to their buoyant mass. Both Au-Tracer@PS and Au-Tracer@PMMA were analyzed by Sed-FFF with the results presented in the supporting information (Supplementary Table S8 and Supplementary Figure S7).

### Chemical Characterization of NP-Tracer Particles

**FIGURE 3:**
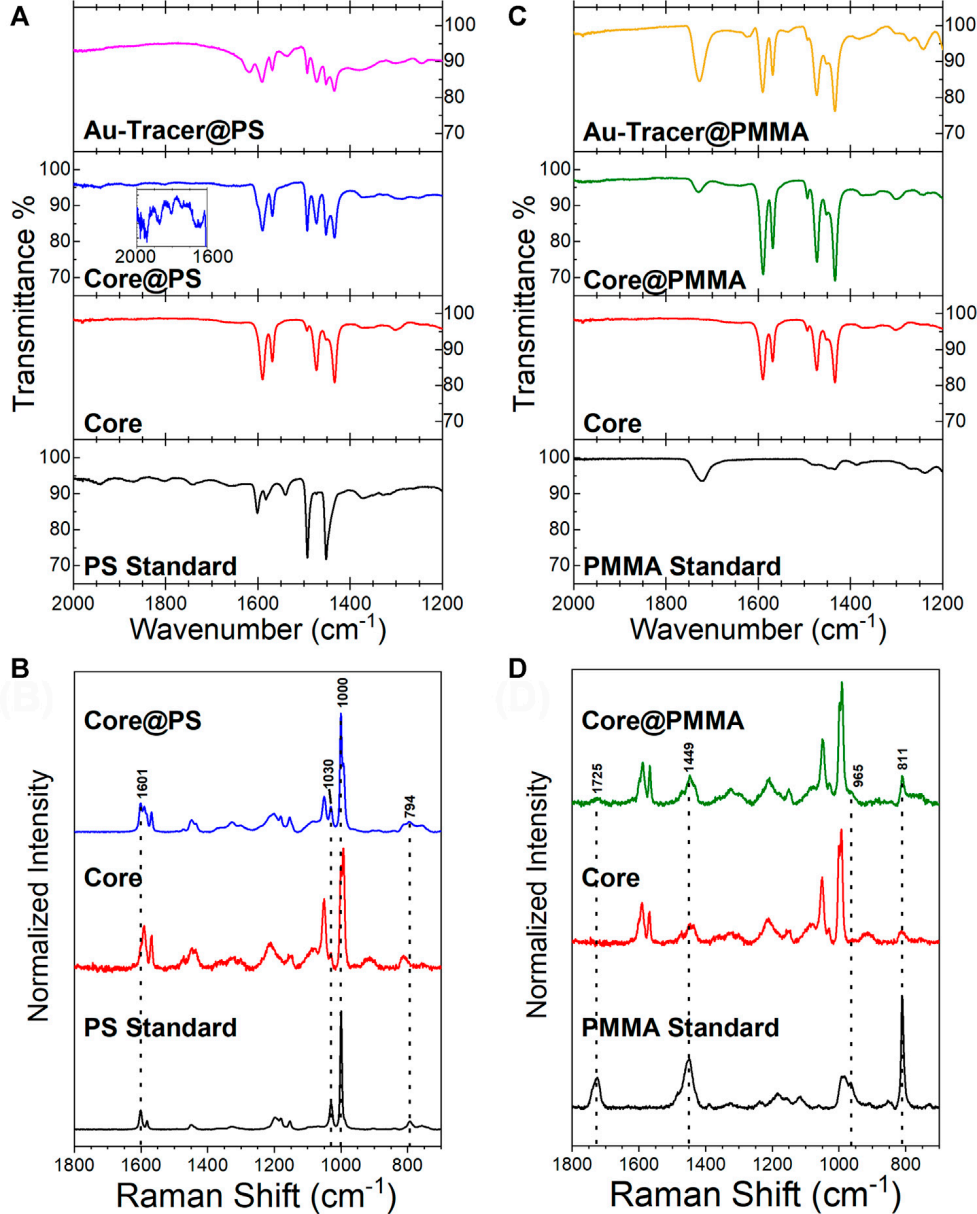
Characterization of Au-Tracer@PS and Au-Tracer@PMMA particles by vibrational spectroscopy. **(A)** FTIR spectra for Core, Core@PS, and Au-Tracer@PS particles in comparison to a PS polymer standard. **(B)** Raman spectra of Core and Core@PS particles in comparison to a PS standard. **(C)** FTIR spectra for Core, Core@PS, and Au-Tracer@PMMA particles in comparison to a PMMA polymer standard. **(D)** Raman spectra of Core and Core@PMMA particles in comparison to a PMMA standard.

The chemical properties of the material were characterized by a number of techniques at each stage of the Tracer synthesis. Figures 3A,B are representative FTIR and Raman spectra for the Core, Core@PS, and Au-Tracer@PS and PS reference material, while Figures 3C,D are representative FTIR and Raman spectra for the Au-Tracer@PMMA and reference PMMA material. A table of identified Raman shifts and IR peaks are presented in the supporting information (Supplementary Tables S9, S10). Additional TGA measurements are presented in the supporting information (Supplementary Figure S8).

### Application of Single Particle ICP-MS to NP-Tracer Characterization

**FIGURE 4:**
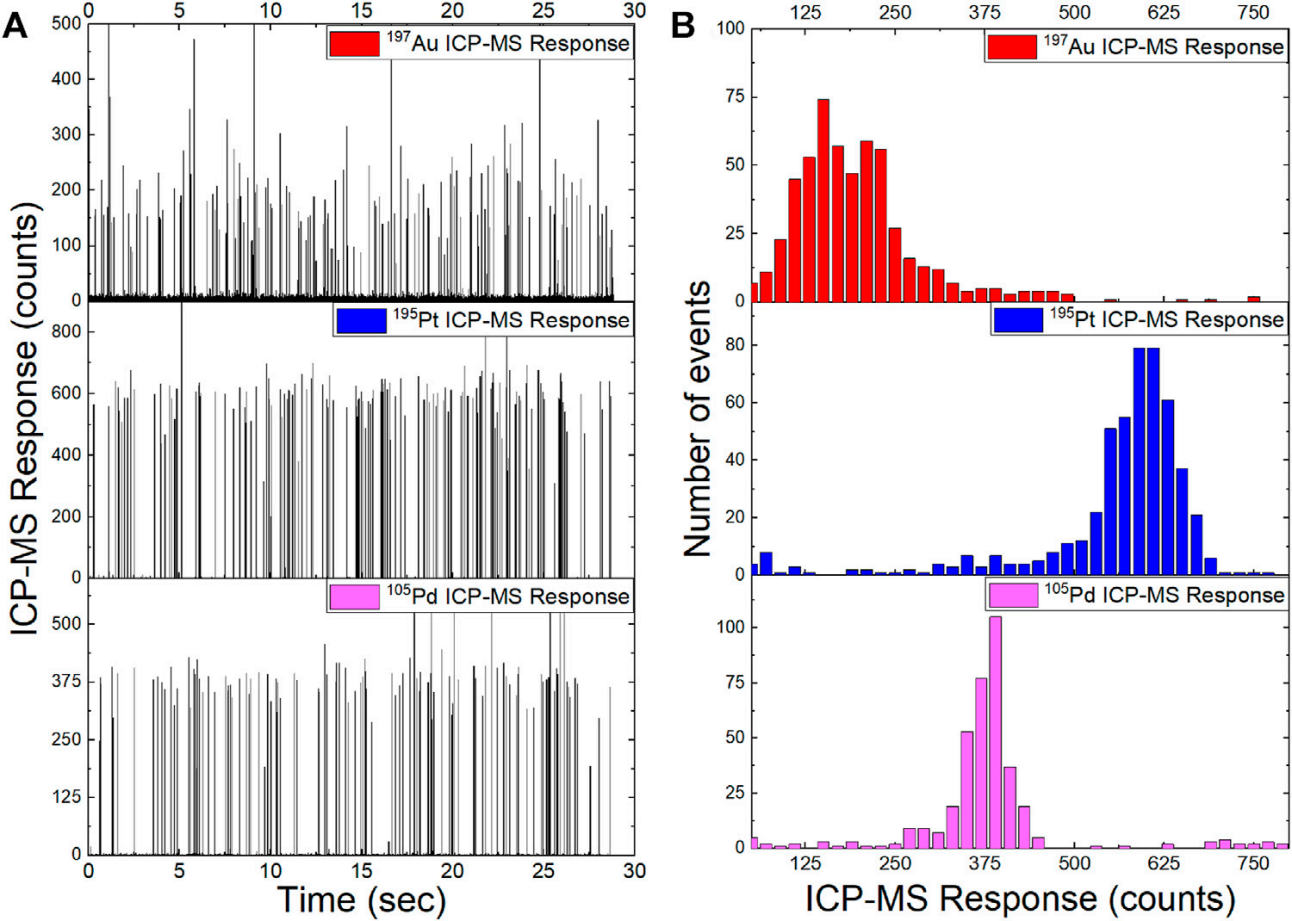
Single particle ICP-MS for Au-Tracer@PS, Pt-Tracer@PS, and Pd-Tracer@PS particles. **(A)** Time trace of raw intensities for the three different metal-NP loaded tracers. **(B)** Histograms of particle intensities for metal-NP loaded tracer particles.

Au-Tracers were analyzed by single particle ICP-MS as shown in Figure 3. To demonstrate the potential for versatility of this tracer platform, three different nanoparticle loadings (Au, Pd, and Pt) were analyzed at a concentration of approximately 10^5^ particles ml^−1^ and representative time traces shown in Figures 4A–C respectively. The height of the peaks represents the mass of metallic nanoparticles tracer embedded within each NP-Tracer. Histograms of the particle intensities demonstrate the reproducibility of the mass loading, as intensity is related to mass and therefore demonstrative of a narrow mass distribution of metal within the particle suspensions. Triplicates of the spICP-MS analysis for the Pt- and Pd-Tracer@PS particles are shown if Supplementary Figures S9, S10. Though only Au-Tracers were used throughout this study, these spICP-MS results show potential promise in that this Core-shell platform may be used to host different metals that may potentially be used to designate different polymer types.

### Environmental Application of NP-Tracers

**FIGURE 5:**
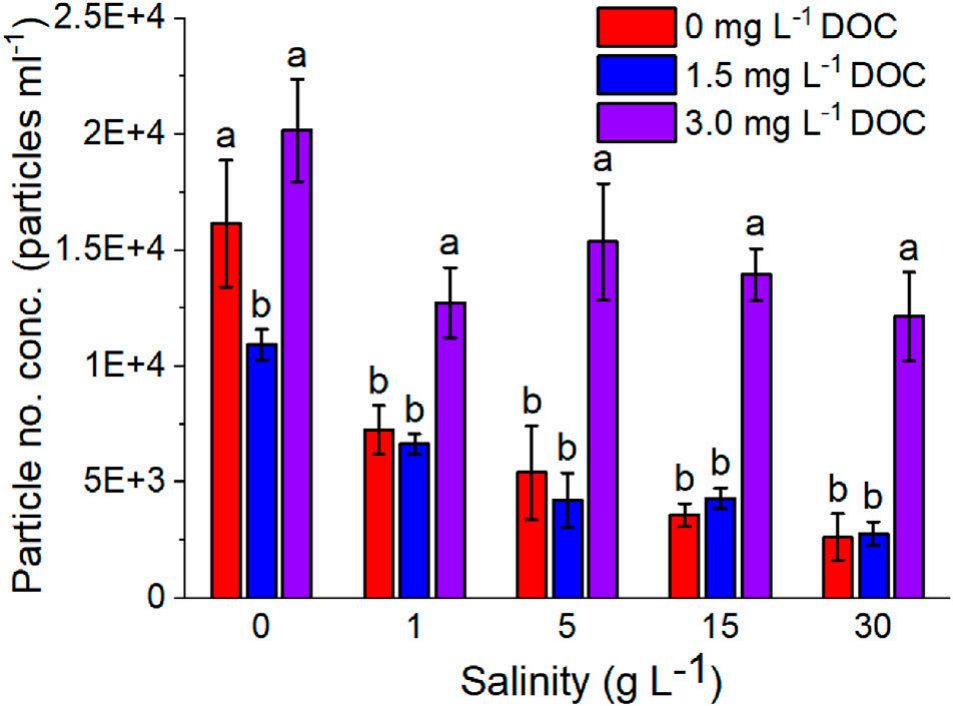
Measured particle number concentrations of Au-Tracer@PS particles in solutions of variable salinity and DOC (SRHA) content (*n* = 3).

The aggregation behavior of the Au-Tracer@PS particles were studied in two different aquatic matrices, EPA moderately hard water (EPA MHW) and 30 g L^−1^ Instant Ocean which had a measured pH value of 7.81 ± 0.01 and 7.23 ± 0.01 respectively. In addition, the settling behavior in the presence of increasing concentrations of dissolved organic carbon in the form of Suwannee river humic acid (SRHA) (measured pH values presented in Supplementary Table S11). Figure 5 shows the measured particle number concentrations as determined by spICP-MS for Au-Tracer@PS particles in increasing salinity and dissolved organic carbon (DOC) concentrations. ANOVA was performed to determine statistically significant differences and a Post-hoc analysis was performed with Tukey’s HSD to determine groups (significance level α = 0.05).

**FIGURE 6:**
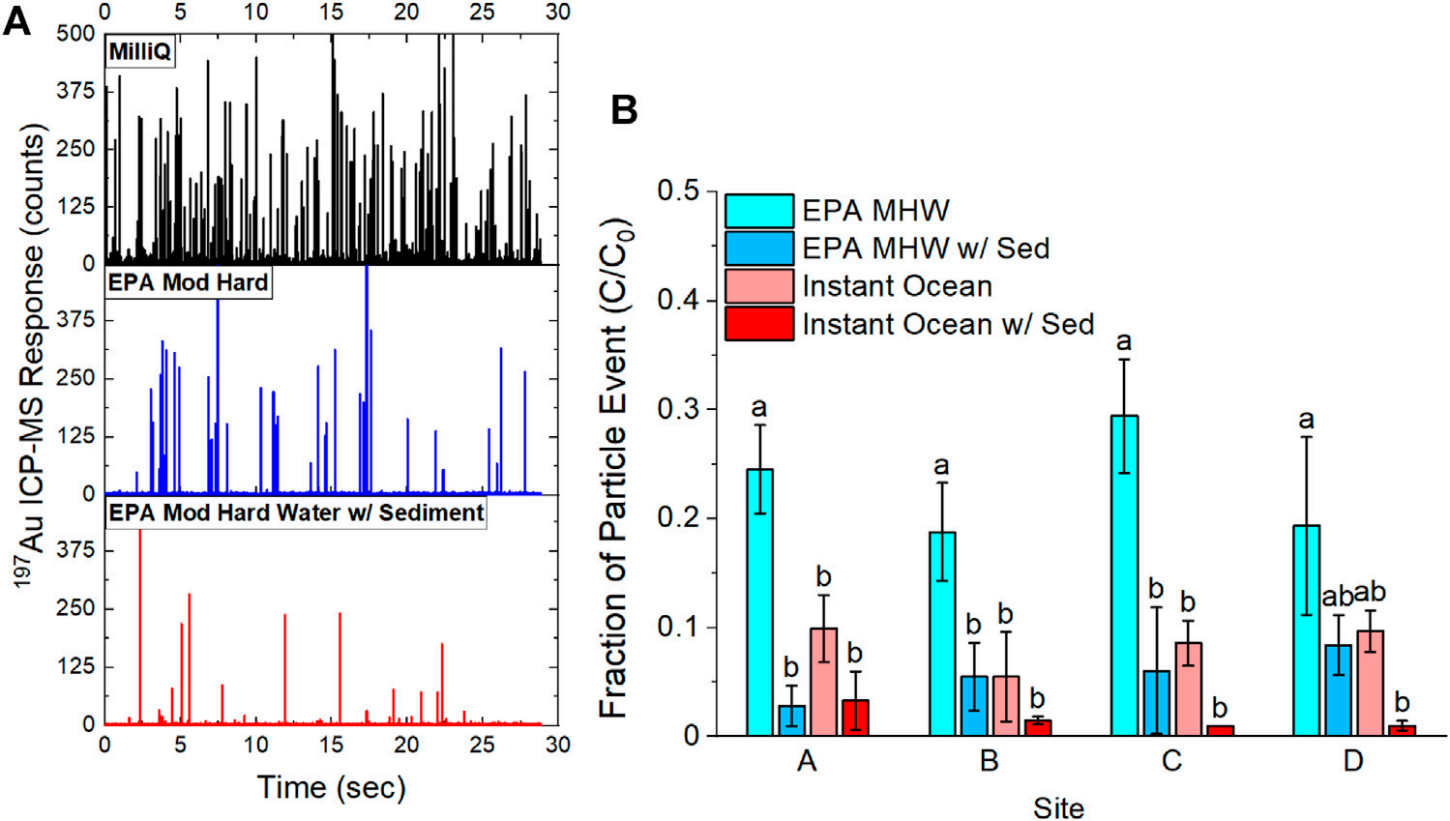
Comparison of Au-Tracer@PS particle events before and after equilibration with sediment slurry. **(A)** Raw date time traces showing Au-Tracer@PS particles in suspension with MilliQ water, EPA moderately hard water, and EPA moderately hard water with sediment. **(B)** Comparison of particle events normalized to particle events in MilliQ water (C_0_) in a freshwater matrix (EPA MHW) and a saltwater matrix (30 g L^−1^ Instant Ocean) with and without the addition of sediment (*n* = 3).

Particle aggregation and settling behavior was also studied in the presence of estuarine sediment collected from Bellingham Bay, WA, United States. Au-Tracer@PS particles were suspended in a sediment slurry and equilibrated over a 48 h time period with continuous shaking. Suspensions were then allowed to settle for 40 min such that the largest material would settle according to stokes’ settling law. Aliquots were then pipetted from the top 2 cm of the solution and analyzed by spICP-MS. A visual representation of the differences in spicp-ms data for Au-Tracer@PS particles in ultrapure water (MilliQ), EPA MHW, and EPA MHW with sediment is shown in Figure 6A. The results shown in Figure 6B detail the fraction of particle events in the different treatments (EPA MHW, Instant Ocean, with and without sediment) using sediment collected from different sites within Bellingham Bay. ANOVA was performed to determine statistically significant differences and a Post-hoc analysis was performed with Tukey’s HSD to determine groups (Significance Level α = 0.05)

### Discussion

#### Polymer Synthesis Enables Mono-Disperse Nanoplastic Suspensions

In this work, we successfully demonstrate that PS and PMMA can be added as a shell to the PS-co-P2VP core particle by seeded emulsion polymerization of methylmethacrylate or styrene (top and bottom rows in Figure 2, respectively). The application of PMMA and PS to a PS-co-P2VP core results in measurable diameter difference as determined by SEM, AFM and DLS (Figure 2C, respectively). The hydrodynamic diameter (D_h_) of the PS-co-P2VP core was determined to be 367 nm by DLS and increased to 441 and 404 nm when PS and PMMA exterior polymer shells are applied (Table 1). Low pH (pH ∼ 2.5) loading of AuCl_4_^−^ into the PS-co-P2VP core and subsequent photoreduction further tunes the DLS-determined D_h_ values to 446 nm (bottom panel Figure 2C) and 494 nm, respectively. Reported in Figure 2A is the STEM image of the resulting Au NP-Tracer@PS where brighter Au NPs located in the core are visible and an exterior PS shell ∼15–20 nm thick can be identified. AFM of a quasi-hexagonally packed monolayer of the Au NP-Tracer@PS is depicted in Figure 2B. Such uniform packing is typical of monodisperse and uniformly shaped particles, where, a diameter of 480 nm for the particles was calculated from the lattice in this AFM image. As evident in these STEM and AFM images, as well as through inspection of the standard deviation values (Table 1), the synthetic approach generates well-defined and monodisperse materials that will permit greater sensitivity quantification when applied to environmental monitoring experiments. Additional evidence for the inclusion of the PS and PMMA shell is provided in the TGA analysis where the non-zero mass percent plateaus that result for Au NP-Tracer@PS and Au NP-Tracer@PMMA (Supplementary Figure S7) are lower than analogues lacking the PS and PMMA shell (Curtis et al., 2018).

Additional physical characterization was provided by field flow fractionation (Supplementary Figure S7). Asymmetric flow field flow fractionation (AF4) separates particles according to their diffusion coefficient which is related to their hydrodynamic diameter. In normal mode, smaller particles will elute the earliest being subject to higher velocity flow lines in the parabolic flow profile (Giddings, 1993; Giddings, 1995). Sedimentation field flow fractionation (Sed-FFF) uses a centrifugal force instead of a cross-flow to force particles towards the accumulation wall; separating particles according to their buoyant mass, where less dense particles elute earliest (Gimbert et al., 2005; Tadjiki et al., 2017). Taken together these techniques provide information on both the particle size (AF4) and particle density (Sed-FFF). Sed-FFF was used to examine the density of both the Au-Tracer@PMMA and Au-Tracer@PS particles, as particle density plays an important role in the settling velocity of suspended particles (Waldschlager and Schuttrumpf, 2019). The measured densities for both particle types were higher than standard polystyrene and PMMA particle densities (Supplementary Figure S7 and Supplementary Table S8) demonstrating some need for improvement in the development of these tracers in order to achieve better agreement between the tracer material and the intended physical properties of the nanoplastic. However, experimental measurements have also shown that the density of microplastics in the environment is highly variable due to weathering and the formation of surface biofilms that can have outsized effects on the particle settling behavior (Kane and Clare, 2019; Waldschlager and Schuttrumpf, 2019; Waldschlaeger et al., 2020). As the intent of these experiment was to demonstrate the broad applicability of the tracer platform and its adsorption to estuarine sediment, this difference was noted as a potential area for future research that may be mitigated by the selection of a less dense metal NP and/or a reduced loading of metal content. One additional application of these materials is to address the impact of weathered polymer nanocomposites (PNCs); polymeric materials that have embedded nanomaterials to imbue them with specific properties. Work has been performed in this area (Barber et al., 2020), utilizing spICP-MS to track gold nanoparticles in polymer films, this application might also be applied to this core-shell platform enabling the estimation of polymer fragmentation during use.

#### Core-Shell Platform Allows for Tracers of Various Polymer Compositions

Core@PMMA and Core@PS were characterized for chemical composition and structure using IR and Raman spectroscopy. The FTIR spectra of the polymer cores show characteristic absorbance bands for polymers of each included monomer, with peaks from PS and P2VP emphasized. Figure 2A depicts a portion of the full FTIR spectra of the polymer Cores, Core@PS, and Au-Tracer@PS (PS spectrum provided as reference). The IR peak at 1,601 cm^−1^ (ring stretch) is assigned to PS with characteristic overtones observed between 1,600 – 1950 cm^−1^ (Silverstein et al., 1981; Mark, 1999), and IR peaks at 1,591, 1,473, and 1,425 cm^−1^ (C=C and C=N ring-stretching) are assigned to P2VP. Characteristic Raman bands are also observed for PS and P2VP in both the Core and Core@PS spectra shown Figure 3B. Prominent PS Raman peaks appear at to 1,601 cm^−1^ (ring stretch), 1,030 cm^−1^ (CH in plane bend), 1,000 cm^−1^ (ring breathing), and 794 cm^−1^ (C=C stretch of ring and backbone) (Tsai et al., 1991). P2VP Raman peaks appear at 992 cm^−1^ (ring breathing) and 1,590 cm^−1^ (ring stretch) respectively (Tsai et al., 1991). For the Core@PS particles, characteristic IR and Raman bands correspondingly increase for PS, due to the increased thickness of the PS shell coating.

The FTIR and Raman spectra and peak assignments of the polymer cores shown in Figures 3C,D are consistent with those discussed for Figures 3A,B. Figure 3C shows a PMMA reference IR spectrum with characteristic bands near 1720 cm^−1^ (C=O stretch) and 1,240 cm^−1^ (C-O stretch) respectively (Lipschitz, 1982; González-Benito and González-Gaitano, 2008). In Figure 3D, the PMMA reference exhibits Raman peaks at 1725 (C=O stretch), 1,449 cm^−1^ (O-CH_3_ bending), 965 cm^−1^ (CH_2_ wagging), and 811 cm^−1^ (C-O-C stretch) (Chen et al., 2019). Appearance of these characteristic PMMA peaks in the Core@PMMA IR and Raman spectra is consistent with the formation of a PMMA shell around the polymer core.

FTIR spectra of the Au-Tracer@PS (Figure 2A) and Au-Tracer@PMMA (Figure 3A) show only minimal changes in the vibrational spectra upon gold ion-loading and subsequent photoreduction to Au NPs. Obtaining Raman spectra of the Au-Tracer@PS and Au-Tracer@PMMA was not deemed feasible because of photo-induced damage of the material caused by the excitation laser.

#### Broad Versatility of NP-Tracer Platform

The spectroscopic data in Figure 3 details the potential to ‘shell’ the core material with a desired polymer such that the environmental matrix interacts with specificity toward the polymer composition. The PS-co-P2VP cores have successfully been modified to include PS and PMMA and we expect that other vinylic-sourced polymers (e.g., butyl rubber, natural rubber, vinylchloride, etc.) can easily be incorporated in future studies. Additionally, the ability to adjust the hydrophilicity of the cores via adjustment of the pH of the solution may allow for inclusion of more hydrophilic polymers as the shell material. Moreover, this platform also allows for different metal nanoparticle signatures to be used to differentiate between different tracer types. As Figure 4 shows: platinum (Pt), palladium (Pd), and gold (Au) can be readily photoreduced to produce metallic NP signatures that can be used for multiplexed detection and identification, which enables tracking of the tracer material by spICP-MS for different polymer types at the single particle level. The relatively narrow intensity distribution shown in Figure 4B and mass and size distributions shown in Supplementary Figures S11, S12 demonstrate that monodispersity of the metal NP loading and the potential to expand on this platform for future environmental studies.

### Metal NP Tracers Enable Single Particle Environmental Studies

The use of metallic NP tracers within micro- and nano-plastics has shown great utility in assessing the environmental fate, transport, and behavior of plastic pollution (Mitrano et al., 2019; Schmiedgruber et al., 2019; Frehland et al., 2020). The use of rare metals as tracers, in particular, allows for easily detectable micro- and nano-plastic particles in complex environmental and biological matrices. We examined the settling behavior of the Au-Tracer@PS particles as a demonstration of the potential utility of this system in assessing micro- and nano-plastic behavior in environmental matrices. The Au-NP tracer was among the first metals used given its relative inertness and monoisotopic nature (as opposed to palladium and platinum), enabling adequate sensitivity for ICP-MS detection. It is possible to further reduce the mass loading to the appropriate detection level for spICP-MS (Lee et al., 2014), however it was kept above this limit to reduce the potential of false negatives in the study. The PS coating was used given the ubiquity of polystyrene pollution and debris relative to PMMA (Erni-Cassola et al., 2019; Jones et al., 2020).

#### Settling Behavior in Aquatic Environmental Matrices

Studying the settling behavior in both freshwater and saltwater matrices is important as estuaries are a likely sink for microplastic pollution in the environment (Wu et al., 2019; Xu et al., 2020). Colloidal stability is highly dependent on ionic strength, such that the rapid change from a freshwater system to saltwater results in particle instability, subsequent aggregation, and settling at the estuary interface (Lasareva et al., 2017).

Figure 5 demonstrates this behavior as we examine the settling behavior of the Au-Tracer@PS particles from 0 – 30 g L^−1^ Instant Ocean. Instant Ocean is a commercial mixture used in aquariums to mimic the salt content of a typical ocean sample. Examining the range of salinities allows the study of particle settling behavior as they migrate from freshwater to saltwater regimes.

Consistent with DLVO theory (Derjaguin and Landau, 1993; Segets et al., 2011) and existing literature examining microplastic behavior, we see a decrease in particle number concentration with increasing salinity. The same behavior was examined in the presence of increasing dissolved organic carbon (Figure 5, DOC in the form of SRHA) and it was apparent that in the intermediate DOC concentration (1.5 mg L^−1^) there was an increase in the settling of the Au-Tracer@PS particles, likely due to divalent cation bridging (DCB) (Sobeck and Higgins, 2002; Wall and Choppin, 2003). However, at higher concentrations of DOC (3.0 mg L^−1^), the particles were stabilized which might be brought by electrostatic or steric forces prevent particle aggregation (Diegoli et al., 2008; Stankus et al., 2011; Nason et al., 2012). Zeta potential measurements (Supplementary Table S12) indicate that the particle surface charge decreased toward zero and increasing salinities, brought on by electrical double layer compression, and also decreased with increasing DOC addition through charge neutralization. Though particle surface charge was near-neutral at the highest DOC concentration (3.0 mg L^−1^) their apparent stability could be explained by the humic acid imparting steric stability, preventing particle aggregation and subsequent settling.

#### Settling Behavior in Estuarine Sediment Systems

To further expand on the work with aquatic media, sediment slurries were prepared with sediment collected from Bellingham Bay in both moderately hard water (EPA MHW) and 30 g L^−1^ Instant Ocean (Schierz et al., 2012; Montano et al., 2020). Figure 6A shows a comparison of the raw time traces where in the fresh MilliQ system (no sediment), there is an abundance of particle events that decrease as we move to a solution of higher ionic strength (EPA MHW, no sediment) and then decreases further as we move to a solution containing sediment (EPA MHW, w/sediment). Figure 6B shows the fraction of particle events, represented as the number of events (C) divided by the number of events in a fresh suspension of tracer particles (C_0_). It is evident that higher ionic strength leads to greater settling in the absence of sediment, when comparing EPA MHW and Instant Ocean suspensions. The presence of sediment further reduces the particle concentration as much as 95% of the initial particle concentration; likely a consequence of the Au-Tracer@PS particles binding to sediment particles that then settle out of suspension (Kowalski et al., 2016).

Slight differences were observed between sites, but none that were statistically significant. It is possible that with a more varied sediment composition between slurries, significant differences may arise between the settling behavior of the Au-Tracer@PS particles.

## Conclusion and Future Work

To better understand the risks of micro- and nanoplastic pollution on the environment and human health, a multi-faceted approach will be necessary to understand the scope of this pollution. The versatile NP-tracer@polymer materials described in this work provide a platform for the study of a range of environmentally relevant micro- and nanoplastics as well as the ability to incorporate elements that enable the application of some of the most sensitive analytical mass spectroscopy techniques (i.e., ICP-MS and spICP-MS). As more sensitive and sophisticated analytical methods for micro- and nano-plastic monitoring continue to develop, the use of tracers in laboratory and artificial environments can provide insight into micro- and nano-plastic behavior. The exploitation of metal-based tracer via monitoring with sensitive instrumentation may offer a way forward in examining the uptake, distribution, and ultimate fate of MPs. Moreover, significant gains in ICP-MS instrumentation, such as ICP-TOF-MS (Hendriks et al., 2017; Praetorius et al., 2017; Naasz et al., 2018) can allow for multi-metal single particle detection, further enabling multiplexed microplastic tracer technologies. Despite existing hurdles impeding better agreement between tracer properties and their intended corresponding polymer, future work examining different metal NP materials might be key to addressing these obstacles.

## Data Availability

Data will be made available by the authors upon request.
