# Supplementary material for: Development and Application of Nanoparticle-Nanopolymer Composite Spheres for the Study of Environmental Processes
Source: Front Toxicol. 2021 Dec 13;3:752296. doi: 10.3389/ftox.2021.752296 (PMC8915914; doi:10.3389/ftox.2021.752296)
Supplement: Supplementary file 1 [file DataSheet1.docx]

Supplementary Material

Development and application of Au Nanoparticle-nanopolymer composite spheres for environmental monitoring

Robert J. Rauschendorfer^1^, Kyle M. Whitham^2^, Star Summer^2^, Samantha A. Patrick^2^, Aliandra E. Pierce^2^, Haley Sefi-Cyr^1^_,_ Soheyl Tadjiki^3^, Michael D. Kraft^5^, Steven R. Emory^2^, David A. Rider^2,4^, Manuel D. Montaño^1,*^

^1^Department of Environmental Sciences, Western Washington University, Bellingham, WA, United States of America

^2^ Department of Chemistry, Western Washington University, Bellingham, WA, United States of America

^3^Postnova Analytics Inc., Salt Lake City, UT, United States of America

^4^Department of Engineering and Design, Western Washington University, Bellingham, WA, United States of America

^5^Scientific Technical Services, Western Washington University, Bellingham, WA, United States of America

**Table S1** Operating Conditions AF4.

**Table S2** Operating Conditions Sed-FFF for Au-Tracer@PS Analysis.

**Table S3** Operating Conditions Sed-FFF for Au-Tracer@PMMA Analysis.

**Table S4** Acquisition Parameters for Raman Microscope**.**

**Table S5** Typical running instrument conditions for ICP-MS.

**Figure S1** Comparison of digested Au-Tracer@PS stocked post filtration

**Figure S2** Sampling map of Bellingham Bay

**Table S6** Carbon and metal content in collected sediment

**Table S7** Measured sediment grain size (presented as wt%)

**Figure S3** Sediment grain size by weight %.

**Figure S4** DLS measurement of (A) Core material (B) Core@PMMA and (C) Au-Tracer@PMMA.

**Figure S5** Measured particle number concentration of Au-Tracer@PS particle suspension at different dilutions.

**Figure S6** SEM-EDS imaging of Au-Tracer@PS,

**Figure S7** AF4 and Sed-FFF fractograms of Au-tracer@PS material.

**Table S8** Calculated particle densities from Sed-FFF.

**Table S9** Raman Band Assignments for Core@PS.

**Table S10** Raman Band Assignments for Core@PMMA.

**Figure S8** TGA plots for components of Au-Tracer@PS and Au-Tracer@PMMA.

**Figure S9** Histogram distribution of Pt-Tracer@PS particles.

**Figure S10** Histogram distribution of Pd-Tracer@PS particles.

**Table S11** Measured pH values for DOC, salinity aggregation experiments.

**Table S12** Zeta potential values (measured in mV) of Au-Tracer@PS particles

**Figure S11** ^197^Au Mass distribution of Au-Tracer@PS particles.

**Figure S12** Equivalent diameter of ^197^Au Mass of Au-Tracer@PS particles.

**Table S1.** Operating Conditions AF4.

| **AF4 (Postnova, AF2000)** | |
| --- | --- |
| **Instrument Parameter** | **Value** |
| Channel Flow Rate | 1 mL min^-1^ |
| Cross Flow Rate (Initial / Final) | 0.5 mL min^-1^ / 0.1 mL min^-1^ |
| Cross Flow Decay Type (Exponent) | Power (0.5) |
| Pre-decay period / Decay Period | 2 min / 35 min |
| Injection flow rate | 0.2 mL min^-1^ |
| Injection/focusing Time (Focus flow rate) | 15 min (1.3 mL min^-1^) |
| Injection Volume | 20 μL |
| Membrane | 10 kDa Regenerated Cellulose |
| Spacer | 350 μm |
| Carrier Solution | 0.05% (v/v) FL-70 + 3 mM NaN_3_ |

**Table S2.** Operating Conditions Sed-FFF for Au-Tracer@PS Analysis.

| **Sed-FFF (Postnova, CF2000)** | |
| --- | --- |
| **Instrument Parameter** | **Value** |
| Field | 1000 RPM |
| Channel Flow Rate | 2 mL min^-1^ |
| Relaxation Time | 5 min |
| Channel Thickness | 131 μm |
| Injection Volume | 50 μL |
| Carrier Solution | 0.05% (v/v) FL-70 + 3 mM NaN_3_ |

**Table S3.** Operating Conditions Sed-FFF for Au-Tracer@PMMA Analysis.

| **Sed-FFF (Postnova, CF2000)** | |
| --- | --- |
| **Instrument Parameter** | **Value** |
| Field | 300 RPM |
| Channel Flow Rate | 1 mL min^-1^ |
| Relaxation Time | 10 min |
| Channel Thickness | 131 μm |
| Injection Volume | 100 μL |
| Carrier Solution | 0.05% (v/v) FL-70 + 3 mM NaN_3_ |

**Table S4.** Acquisition Parameters for Raman Microscope**.**

| **Parameter** | **Setting** |
| --- | --- |
| Objective | Leica N Plan 50×L, NA = 0.50 |
| Laser & Excitation Wavelength | HeNe, 632.8 nm |
| Spot Size on Sample | 1.3 μm |
| Laser Power at Sample | 5.0 mW |
| Power Density | 3.8 mW/μm^2^ |
| Integration Time | 10 s |
| Spectra Averaged per Sample | 12 |

**Table S5** Typical running instrument conditions for ICP-MS.

| **ICP-MS (Agilent 7500ce)** | |
| --- | --- |
| **Instrument Parameter** | **Value** |
| Nebulizer Gas Flow | 0.81 ml/min |
| Sample Flow Rate | 0.34 ml/min |
| Spray Chamber | Scott Double Pass |
| ICP RF Power | 1500 W |
| Dwell time | 10 ms |
| Transport efficiency | 4-6% |
| Analytes | ^197^Au, ^105^Pd, ^195^Pt |
| Analysis Time | 30 s |





**Figure S1** Comparison of digested Au-Tracer@PS stocked post filtration with 0.22 μm cellulose syringe filter in three different media. Measurements were taken from a freshly-prepared stock and one equilibrated for 48hrs.

**
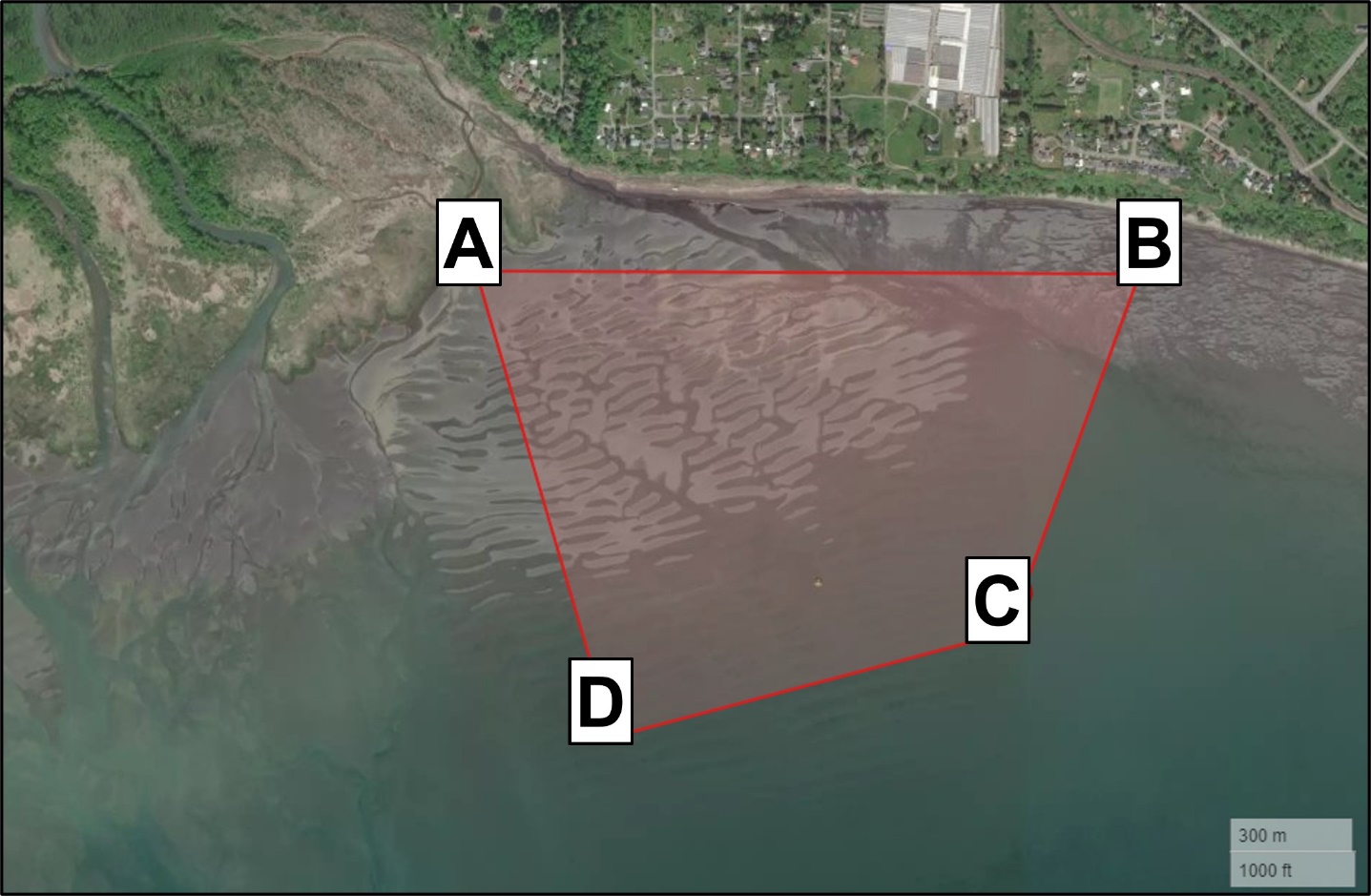
**

**Figure S2** Sampling map of Bellingham Bay (*Source: GeoJSON open source mapping software*).

**Table S6** Carbon and metal content in collected sediment

| **Site** | **TC Conc. (g/kg)** | **BC Conc. (g/kg)** | **Mn Conc. (mg/kg)** | **Ni Conc. (mg/kg)** | **Cu Conc. (mg/kg)** | **Zn Conc. (mg/kg)** | **Al Conc. (g/kg)** | **Fe Conc. (g/kg)** | **Mg Conc. (g/kg)** | **Ca Conc. (g/kg)** |
| --- | --- | --- | --- | --- | --- | --- | --- | --- | --- | --- |
| A | 1.4 ± 0.1 | 0.07±0.01 | 451 ± 6 | 129 ± 15 | 26 ± 1 | 44 ± 2 | 22 ± 2 | 61 ± 6 | 56 ± 5 | 15 ± 2 |
| B | 1.6 ± 0.2 | 0.07±0.03 | 267 ± 11 | 121 ± 18 | 24 ± 3 | 42 ± 2 | 22 ± 3 | 62 ± 4 | 55 ± 6 | 17 ± 1 |
| C | 6.5 ± 0.5 | 0.07±0.01 | 319 ± 11 | 99 ± 1 | 40 ± 3 | 58 ± 1 | 30 ± 2 | 69 ± 1 | 48 ± 0.4 | 17 ± 2 |
| D | 2.2 ± 0.1 | 0.09±0.02 | 350 ± 5 | 118 ± 4 | 27 ± 4 | 48 ± 1 | 24 ± 3 | 62 ± 5 | 53 ± 3 | 17 ± 0.2 |

**Table S7** Measured sediment grain size (presented as wt %)

| **Site** | **Percent Coarse Sand (2000-500µm)** | **Percent Medium Sand (500-250µm)** | **Percent**  **Fine Sand**  **(250-125µm)** | **Percent**  **Very Fine Sand**  **(125-63 µm)** | **Percent**  **Silt**  **(<63µm)** |
| --- | --- | --- | --- | --- | --- |
| A | 0.52 ± 0.07 | 13.77 ± 1.97 | 72.46 ± 1.35 | 11.43 ± 2.35 | 1.83 ± 0.76 |
| B | 2.55 ± 1.19 | 25.95 ± 4.36 | 51.19 ± 12.39 | 15.87 ± 9.12 | 4.40 ± 3.39 |
| C | 0.82 ± 0.34 | 0.96 ± 0.39 | 1.80 ± 0.25 | 16.84 ± 1.56 | 79.54 ± 1.04 |
| D | 0.41 ± 0.02 | 6.02 ± 0.37 | - 1. ± 1.01 | 48.52 ± 1.00 | 10.18 ± 0.36 |

**Figure S3** Sediment grain size by weight %.

**Figure S4** DLS measurement of (A) Core material (B) Core@PMMA and (C) Au-Tracer@PMMA particles.

**Figure S5** Measured particle number concentration of Au-Tracer@PS particle suspension at different dilutions (n=3) as determined by spICP-MS. The steady decrease at higher concentrations (lower dilution factors) is likely a consequence of ‘coincidence’ where high particle concentrations result in overlapping signals upon detection.





**Figure S6** SEM-EDS imaging of Au-Tracer@PS,

**

**

**Figure S7** FFF fractograms of Au-tracer@PS material. (A) SedFFF-UV-vis fractogram of Au-tracer@PS particle suspension in comparison to a 512 polystyrene particle standard. (B) AF4-UV-vis fractogram of Au-tracer@PS particle suspensions in comparison to a 512 polystyrene and 600nm polystyrene particle standard.

**Table S8** Calculated particle densities from Sed-FFF.

| **Sample** | **Calculated density(kg/m^3^)** |
| --- | --- |
| 512 nm Polystyrene standard | 1045 |
| 499 nm Polymethylmethacrylate standard | 1164 |
| Au-Tracer@PS | 1135 |
| Au-Tracer@PMMA | 1210 |

**Table S9** Raman Band Assignments for Core@PS.

| **Core** | | **Core@PS** | |
| --- | --- | --- | --- |
| **Raman Shift (cm^-1^)** | **Assignment** | **Raman Shift (cm^-1^)** | **Assignment** |
| 1601 (shoulder) (m) | PS ring stretch | 1601 (s) | PS ring stretch |
| 1590 (s) | P2VP ring stretch | 1590 (m) | P2VP ring stretch |
| 1567 (m) | PS ring stretch | ----- | ----- |
| ----- | ----- | 1568 (m) | PS ring stretch |
| 1447 (m) | P2VP ring stretch | 1447 (m) | P2VP ring stretch |
| 1330 (w) | PS/P2VP CH2 twist |  |  |
| ----- | ----- | 1328 (w) | PS/P2VP CH2 twist |
| 1210 (m) | PS C=C of ring & backbone | ----- | ----- |
| ----- | ----- | 1202 (m) | PS C=C of ring & backbone |
| ----- | ----- | 1153 (m) | P2VP CH in plane bend |
| 1149 (m) | P2VP CH in plane bend | ----- | ----- |
| 1087 (w) | P2VP CH in plane bend | ----- | ----- |
|  | ----- | 1051 (s) | PS CH in plane bend |
| 1050 (s) | PS CH in plane bend | ----- | ----- |
| 1030 (w) | PS CH in plane bend | 1030 (m) | PS CH in plane bend |
| 1000 (shoulder) (s) | PS/P2VP ring breathing | 1000 (s) | PS/P2VP ring breathing |
| 992 (s) | P2VP ring breathing | 992 (shoulder) (s) | P2VP ring breathing |
| 914 (w) | P2VP C-C vibrating | ----- | ----- |
| 812 (w) | P2VP C=C of ring & backbone | ----- | ----- |
| ----- | ----- | 794 (m) | PS C=C of ring & backbone |

**Table S10** Raman Band Assignments for Core@PMMA.

| **Core** | | **Core@PMMA** | |
| --- | --- | --- | --- |
| **Raman Shift (cm^-1^)** | **Assignment** | **Raman Shift (cm^-1^)** | **Assignment** |
| ----- | ----- | 1725 (w) | PMMA C=O |
| 1601 (shoulder) (m) | PS ring stretch | ----- | ----- |
| 1590 (s) | P2VP ring stretch | ----- | ----- |
| ----- | ----- | 1588 (s) | P2VP ring stretch |
| 1567 (m) | PS ring stretch | 1567 (s) | PS ring stretch |
| 1447 (m) | P2VP ring stretch | 1447 (m) | P2VP ring stretch |
| 1330 (w) | PS/P2VP CH2 twist | ----- | ----- |
| ----- | ----- | 1327 (w) | PMMA CH2 twist |
| 1210 (m) | PS C=C of ring & backbone | 1210 (m) | PS C=C of ring & backbone |
| ----- | ----- | 1150 (m) | P2VP CH in plane bend |
| 1149 (m) | P2VP CH in plane bend | ----- | ----- |
| 1087 (w) | P2VP CH in plane bend | ----- | ----- |
| 1050 (s) | PS CH in plane bend | 1050 (s) | PS CH in plane bend |
| 1030 (w) | PS CH in plane bend | ----- | ----- |
| ----- | ----- | 1029 (w) | PS CH in plane bend |
| 1000 (shoulder) (s) | PS/P2VP ring breathing | 1000 (shoulder) (s) | PS/P2VP ring breathing |
| 992 (s) | P2VP ring breathing | 992 (s) | P2VP ring breathing &  PMMA O-C rocking |
| ----- | ----- | 965 (shoulder) (w) | PMMA CH2 wagging^3^ |
| 914 (w) | P2VP C-C vibrating | ----- | ----- |
| 812 (w) | P2VP C=C of ring & backbone | ----- | ----- |
| ----- | ----- | 811 (m) | P2VP C=C of ring &  PMMA C-O-C |

**Figure S8** TGA plots for components of Au-Tracer@PS and Au-Tracer@PMMA. To ensure complete volatilization and/or combustion of organic materials the purge gas of the TGA furnace was switched from inert N_2_ to air at a temperature of 650°C. The resulting plateau after this event represents the metal mass percent in each sample.

**Figure S9** Histogram distribution of Pt-Tracer@PS particles showing relative monodispersity of Pt-NP loading

**Figure S10** Histogram distribution of Pd-Tracer@PS particles showing relative monodispersity of Pd-NP loading.

**Table S11** Measured pH values for DOC, salinity aggregation experiments.

| **Salinity conc. (g L^-1^)** | **Dissolved Organic Carbon Conc. (mg L^-1^)** | | |
| --- | --- | --- | --- |
|  | **0** | **1.5** | **3.0** |
| 0 | 6.51 ± 011 | 7.31 ± 0.19 | 7.25 ± 0.06 |
| 5 | *** | 5.39 ± 0.26 | 5.10 ± 0.04 |
| 15 | 6.55 ± 0.09 | 5.35 ± 0.18 | 4.50 ± 0.22 |
| 30 | 6.63 ± 0.34 | 5.21 ± 0.18 | 4.67 ± 0.05 |

**Table S12** Zeta potential values (measured in mV) of Au-Tracer@PS particles in different aquatic media

| **Salinity (g L^-1^)** | **Dissolved Organic Carbon (mg L^-1^)** | | |
| --- | --- | --- | --- |
|  | **0** | **1.5** | **3.0** |
| 0 | 28.6 (±0.5) | 16.4 (±0.7) | 7.9 (±0.1) |
| 1 | 19.5 (±0.4) | --- | --- |
| 5 | 6.8 (±2.3) | -4.76^*^ | -2.14^*^ |
| 30 | -0.7 (±1.2) | -1.06^*^ | -2.8^*^ |

**Figure S11** ^197^Au Mass distribution of Au-Tracer@PS particles as measured by spICP-MS (n=5)





**Figure S12** Equivalent spherical diameter based on ^197^Au Mass of Au-Tracer@PS particles as measured by spICP-MS assuming a spherical geometry and a particle density of 19.3 g cm^-3^ (n=5).
